# Amyloids and protein aggregation

**DOI:** 10.1039/d2sc90225g

**Published:** 2023-06-06

**Authors:** Sara Linse, Tuomas Knowles

**Affiliations:** a Lund University, Biochemistry & Structural Biology Chemical Centre Lund Sweden sara.linse@biochemistry.lu.se; b University of Cambridge, Department of Chemistry Cambridge UK tpjk2@cam.ac.uk

## Abstract

A general discovery in protein science in the past few decades has been the finding that a number of unrelated proteins and peptides all have a marked propensity to form amyloid fibrils *in vivo* and *in vitro*. These structures have become known as the pathological hallmark of some of the most prevalent neurodegenerative diseases. More recently, the process of amyloid formation has been demystified through a number of key mechanistic findings, some of which are highlighted in this themed collection.

## Abstract 2

Sara Linse and Tuomas Knowles introduce the *Chemical Science* themed collection on the topic of amyloids and protein aggregation.

## Graphical abstract

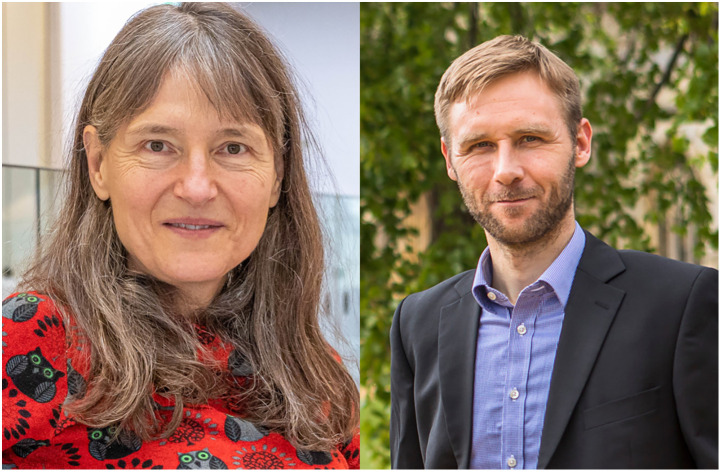

## 1. Equilibrium and solubility

### 1.1 Phase transitions

Amyloid formation from a solution of a peptide or protein is a phase transition which results in highly ordered fibrils in a solid phase that co-exist with a dilute solution phase consisting mainly of monomers and a minority population of oligomers. At equilibrium, the chemical potential of the peptide in solution, *μ*_m_, is the same as for peptides in the fibrillar aggregates, *μ*_f_.^1,2^*μ*_m_ = *μ*^0^_m_ + *RT* ln [*m*] = *μ*_f_ = *μ*^0^_f_

Crucially, the chemical potential of the monomer is concentration dependent, while that of the fibril is not; this situation, therefore, defines quantitatively the equilibrium in terms of the solubility, which is the highest concentration at which the peptide can exist as monomers for infinite time. Above the solubility, the concentration of monomers in the fibril phase grows with monomer concentration while the equilibrium concentration of monomers in solution tends towards a constant. In addition to the solid amyloid phase, dense liquid phases of proteins can form through liquid–liquid phase separation (LLPS,^2–4^). It is becoming increasingly clear that in many cases these functional liquid condensates can promote the formation of pathological amyloid fibrils through undergoing a liquid to solid transition in disease.

### 1.2 Reversibility

Amyloid formation is a non-covalent polymerization reaction and thus in principle reversible without the requirement to break bonds. During the aggregation process, both the forward and backward processes take place, but their relative rates change over time as an equilibrium is being established. This reversibility is readily demonstrated by diluting an equilibrated system, after which some of the fibrils dissolve to establish a new equilibrium defined by the lower total concentration of peptide. Fibrils may also dissolve if brought to solution conditions at which they are less stable than under conditions at which they were formed.

The review by Buell (https://doi.org/10.1039/D1SC06782F) provides a comprehensive summary of the thermodynamics of amyloid formation, highlighting the commonalities and difference between unimolecular protein folding and protein self-assembly. Buell further outlines several examples of methodologies to study amyloid thermodynamics as well as recent findings regarding amyloid stability.

## 2 Experimental challenges and development

If the monomer concentration is brought above the solubility limit, *e.g.* through a temperature jump, the solution becomes supersaturated and the disappearance of monomers and formation of fibrils can monitored over time. Using defined and systematic variations in the starting state it is possible to acquire reproducible data that can form the basis for deriving an aggregation mechanism. The sensitivity of nucleation and growth steps to a range of perturbations including sequence inhomogeneity, additional solution components, surface area and roughness of sample containers, air–water interfaces, *etc.* makes it essential to minimize and gain control over these factors. While initial attempts were focused on pure buffer systems, recent advances have enabled mechanistic insights into aggregation processes *in vivo* (Sinnige, https://doi.org/10.1039/D2SC01278B).

## 3. Kinetics and mechanisms

The thermodynamics of amyloid formation can readily be understood as a simple free energy balance between the dilute solution and the aggregated phase as outlined above. By contrast, it has become clear that the kinetics of this process are remarkably intricate and consist of a number of processes that typically occur simultaneously during the transition. Key amongst these are nucleation processes, which result in the formation of new aggregates from monomeric species, and growth processes, which result in the increase in size of existing aggregates. One key finding from the past ten years is the discovery of secondary nucleation as a key mode of production of new aggregates. Unlike simple nucleation, which is the process by which monomeric peptides come together in solution to form a nucleus of the aggregated phase which can then further grow, secondary nucleation is an aggregate-dependent process, where the surfaces of existing aggregates catalyse the formation of new aggregates. Secondary nucleation is intrinsically a process which is challenging to control since it leads to a runaway reaction where products of the process can catalyse the further conversion of initially monomeric peptides into aggregates. Interestingly, secondary nucleation is emerging as a general feature of disease-associated amyloid formation.

### 3.1 Theory challenges and development

The insights that have emerged in the past ten years from kinetic analysis have generated a mechanistic basis on which to conceptualise amyloid formation. Kinetic analysis has historically been a gold standard tool for validating mechanisms in small molecule chemistry, and these developments suggest that it could be equally valuable in elucidating protein self-assembly and aggregation mechanisms. This work sets the basis for exploring further key mechanistic questions, including the mechanisms of formation and dissociation of oligomers, the connections between liquid condensates and amyloid fibrils and the possibilities and limits of controlling and modulating the aggregation process.

### 3.2 Aggregation mechanism *in vivo*

The perspective by Sinnige (https://doi.org/10.1039/D2SC01278B) discusses efforts towards elucidating the mechanisms of amyloid formation *in vivo* and its link to the emergence of human disease. The perspective highlights the acquisition of quantitative data in the model organisms *Caenorhabditis elegans* and shows how such data can be analysed and interpreted using the same biophysical principles established *in vitro*. Among the challenges discussed are the experimental readout and the high overexpression levels typically used in animal models of protein aggregation diseases. Future efforts may be directed towards understanding the mechanistic influence of chaperones and small-molecule inhibitors. The observations of LLPS at early stages of protein aggregation processes, and their clear existence *in vivo* (for a review see ref. 4), has raised the question of the influence of condensates on for example the nucleation of solid-phase aggregates, in which case nucleation may be either favored or disfavored; examples of the latter are presented by Küffner *et al.* (https://doi.org/10.1039/D0SC04395H).

## 4. Oligomeric intermediates

Analysis of aggregation processes has revealed that they typically result in a very heterogeneous mixture of species of different sizes. Of these, the low relative molecular weight species, oligomers, are particularly reactive in a biological context and are responsible for many of the deleterious effects associated with protein aggregation.^5^ In molecular terms, this may be related to neurotoxic oligomers having distinct surface properties compared to monomers and fibrils, as revealed by high-resolution structural studies (https://doi.org/10.1039/C9SC01331H). Oligomers are often discussed as being either on- or off-pathway, and a recent perspective suggested that on- and off-pathway oligomers may be distinguished by an index that refers to the extent to which an oligomer contributes to amyloid formation (https://doi.org/10.1039/C9SC06501F).

## 5. Role of solution conditions

The sensitivity of aggregation kinetics and equilibria to solution conditions may be seen as a formidable challenge. However, this can also be taken as an advantage in systematic studies of the influence of different types of intermolecular interactions and molecular driving forces. The amyloid self-assembly process is also strongly dependent on factors such as sedimentation, microgravity and air–water interfaces (https://doi.org/10.1039/D0SC00281J). In the total absence of an air–water interface, primary nucleation occurs to a larger extent at other surfaces or in the bulk, in which case a higher degree of hydration of nuclei may affect the aggregate morphology (https://doi.org/10.1039/D0SC05297C). A detailed study of the role of electrostatic interactions revealed a biphasic salt dependence of the scaling exponent, the monomer concentration dependence of the overall aggregation rate (https://doi.org/10.1039/C7SC00215G). The reduction of the scaling exponent at low salt concentration was related to low fibril stability at such conditions, leading to secondary nucleation and fragmentation in parallel. In contrast, the reduction of the scaling exponent at high salt concentration was related to the screening of the repulsion between monomers and between monomers and fibrils such that the detachment of nucleated species becomes rate-limiting for secondary nucleation.

## Supplementary Material
